# Countering Countermeasures: Detecting Identity Lies by Detecting Conscious Breakthrough

**DOI:** 10.1371/journal.pone.0090595

**Published:** 2014-03-07

**Authors:** Howard Bowman, Marco Filetti, Abdulmajeed Alsufyani, Dirk Janssen, Li Su

**Affiliations:** 1 Centre for Cognitive Neuroscience and Cognitive Systems (CCNCS), School of Computing, University of Kent, Canterbury, United Kingdom; 2 Department of Psychology, University of Birmingham, Birmingham, United Kingdom; 3 Helsinki Institute for Information Technology, Aalto University, Helsinki, Finland; 4 Department of Computer Science, Taif University, Taif, Saudi Arabia; 5 NHTV Breda University of Applied Sciences, Breda, The Netherlands; 6 Department of Psychology, Cambridge University, Cambridge, United Kingdom; 7 MRC Cognition and Brain Sciences Unit, Cambridge, United Kingdom; University of Buenos Aires, Argentina

## Abstract

One major drawback of deception detection is its vulnerability to countermeasures, whereby participants wilfully modulate their physiological or neurophysiological response to critical guilt-determining stimuli. One reason for this vulnerability is that stimuli are usually presented slowly. This allows enough time to consciously apply countermeasures, once the role of stimuli is determined. However, by increasing presentation speed, stimuli can be placed on the fringe of awareness, rendering it hard to perceive those that have not been previously identified, hindering the possibility to employ countermeasures. We tested an identity deception detector by presenting first names in Rapid Serial Visual Presentation and instructing participants to lie about their own identity. We also instructed participants to apply a series of countermeasures. The method proved resilient, remaining effective at detecting deception under all countermeasures.

## Introduction

Lie detection is one of the most emotive and hotly debated of all human technological endeavours [1]–[4], with a long, and some would say chequered [5], , history. Prominent lie detection approaches include the standard polygraph [7], which monitors for ‘signature’ changes in (peripheral) autonomic responses, and the, cognitively more central, EEG [8], [9] and, in the last few years, fMRI [10] methods. Amongst the applications of these approaches, detection of identity deception is particularly important [11].

Although a variety of questioning modes exist (e.g. the Control Questions Test [12] and the Guilty Knowledge Test [6]), key to all these approaches is demonstration of a differential response (physiological, electrophysiological or hemodynamic) to a guilt-relevant test when compared to a guilt-irrelevant test. (In the Guilty Knowledge Test, which is our main area of interest, the former of these is often called the Probe and, the latter, the Irrelevant.) However, all such methods are confounded or, at least, significantly complicated by the possibility to apply countermeasures [4], [13]–[15]. For example, Rosenfeld and colleagues developed the Complex Trial Protocol, specifically to prevent countermeasure use. Similarly to previous work [8], their system used the P3 electroencephalographic response to detect deception [9]. However, as demonstrated in later studies by the same group, refined countermeasure strategies allowed that specific deception detection implementation to be partially confounded [16]. Analysis of reaction times (together with careful selection of the number of Irrelevants) and “P9” responses has been argued to discriminate countermeasure users in most cases [14], [17]. It would be desirable, however, to have a deception detection system that prevents P3 amplitudes from being modulated by countermeasures in the first place.

Our proposal responds by presenting critical stimuli on the fringe of awareness, which, we argue, confounds strategies based upon volitional control. A related strategy, in their case fully subliminal, was previously demonstrated by Lui and Rosenfeld [18] (these findings were replicated, somewhat less successfully, in a study that measured skin conductance instead of EEG [19]). We consider how our approach differs from Lui and Rosenfeld’s and their relative success in countering countermeasures in the discussion. Our approach involves, however, Rapid Serial Visual Presentation (commonly abbreviated as RSVP), in which stimuli are presented at the same spatial location at a rapid rate (typically around 10 per second). Since stimuli ‘mask’ one another, the vast majority of RSVP items are not consciously identified [20]. However, stimuli that are salient (whether intrinsically or prescribed by the current task) typically breakthrough into awareness. That is, it seems that during RSVP, the brain is searching for stimuli that are cognitively salient, which, when found, are “presented” to consciousness [21]. We describe the type of cognitive process performed during RSVP as subliminal salience search (SSS) [22] and we call the method we propose the Fringe (or P3-Rapid) identity detector.

Two countermeasure approaches that we tested are, as we call them, “Probes as low-salient” and “Irrelevants as high-salient”. The former of these involves the suspect dampening down the measured response when the Probe is presented, while the latter involves elevating the response measured, e.g. by imagining the questioner hitting them, when an Irrelevant is presented. Importantly, both these countermeasures are reliant upon artificially counteracting the pre-potent neural and bodily response. Such counteraction would be expected to depend upon volitionally applied conscious (cognitive) control.

The method relies upon two particular properties, which we contend are characteristic of the brain’s capacity to subliminally search when overloaded with stimulus processing demands.

1. When subliminally searching, we have little volitional control over breakthrough into conscious awareness. In other words, during such search, conscious access is ballistic; that is, if the subconscious brain detects a salient stimulus, volitional cognitive control cannot “reach down” and stop the “presentation” of that item to the conscious brain.
2. Our perceptual systems are effective at subliminal search for stimuli that are ‘a priori’ salient to us, but much less so (and perhaps not at all) for those that are not salient, although presented frequently.

These two properties map directly onto the proposed countering of countermeasures. Specifically, we argue that the Probes as low-salient strategy is precluded by property 1, while Irrelevants as high-salient is effectively subverted by property 2. The experiments presented here support this position. The key elements, then, of the Fringe/P3-Rapid identity detector are as follows.

Firstly, we present stimuli in RSVP. Secondly, some RSVP streams contain the Probe (the suspected own-name) and some an Irrelevant (a name unknown to the participant, but presented as frequently as the Probe). Importantly, if the Probe were not the suspect’s name, it would be an Irrelevant. Consequently, only true own-name Probes would be differentially processed relative to Irrelevants, and, thus, break into awareness.

Thirdly, we use EEG to detect perceptual breakthrough. Specifically, the P3 Event Related Potential (ERP) component seems only to be present when an RSVP stimulus is consciously perceived [23]–[25]. The P3, then, provides a behavioural report-independent means to determine whether a Probe is perceived and, thus, is salient, as one’s own-name would surely be.

In our experiments, RSVP streams were sequences of first names, each containing one critical item, which was either their own name (the Probe), a name they were asked to pretend was their name (the Fake) or one of two Irrelevants. (Note, the Fake is effectively the target prescribed by the participant’s task, and accordingly, in the literature some do use the term target for what we call the Fake.) We instructed participants to lie and, accordingly, in response to the end of stream question, “Did you see your name?”, they were told to answer “yes” when they saw the Fake and “no” in all other cases. The deception detecting comparison was, then, between the electrical response to Probe and to Irrelevant.

It is important to note that we are not explicitly observing a brain response unique to lying. Rather, our proposal is to piggy-back detection of identity deception upon detection of perceptual breakthrough, which itself is driven by the brain’s (Fringe awareness) detection of salience. Thus, effectively, we turn a perceptual breakthrough detection system (c.f. [22]) into an identity detection system through choice of stimuli and, particularly, the nature of those stimuli’s deception-coloured salience.

## Methods

### 2.1 Deception Detection Experiment

The experimental setting of the deception detector experiments presented here is nearly identical to the original Subliminal Salience Search study [22]. Two differences are present: the delay between the onset of each stimulus (also called the Stimulus Onset Asynchrony (SOA)) and the instructions given to participants. We decreased the SOA between stimuli to 100ms (from the previous 133ms). This increased presentation speed is intended to render countermeasure use less feasible. We also applied a notch filter between 8 Hz and 12 Hz to dampen the 10 Hz oscillations evoked by presenting stimuli every 100ms (in other words, we eliminated, or at least reduced, most Steady-State Visually Evoked Potentials elicited by our RSVP streams). For these reasons, we replicated the previous hit rate and false alarm tests in this study (as experiments number 1 and 5). We gave additional instructions to participants in the remaining experiments (2, 3 and 4) in order to assess countermeasure resilience. These are described in Section 2.1.6. The underlying method that all the experiments described in this paper share with the previous study [22] is an identity deception detector. It can be briefly described as follows.

First names were presented in RSVP trials of 15 items each, one of which was a *critical item*, while the remaining 14 were *distractors.* One of four critical items could appear within each stream: *Probe, Fake, Irrelevant1* or *Irrelevant2.* Probes were the concealed information (participants’ first names), while Fakes were pretend names that participants assumed during the experiment. This was chosen by participants prior to the start of the experiment from a list of 12 possible names, from which they were asked to remove familiar names. The two Irrelevants were randomly selected from the remaining names of the list. They appeared as often as Probes and Fakes over the course of the experiment (50 trials each). The exact structure of the experiment is discussed in Sections 2.1.1–2.1.8. P3 sizes were calculated using Peak-to-Peak differences (the amplitude difference between the highest peak of a waveform and the lowest, where peaks are in fact average amplitudes across short intervals; for details, see Section 2.1.10). We analysed data from three electrodes: Fz, Cz and Pz. We calculated P3a size on Fz and Cz while P3b size was calculated at Pz. Although our use of the term P3a in relation to the fronto-central oscillations we obtain may leave some room for debate, we believe this naming is appropriate (as further justified in the discussion, Section 4.2). We combined data from these three electrodes (Fz, Cz and Pz) using Fisher’s method. Deception probability at the individual level was assessed using a Monte Carlo Permutation Test, also called randomisation, while at the group-level we employed t-tests. For details of these methods, see Sections 2.1.9–2.1.12.

#### 2.1.1 Stimulus Presentation

**Figure 1:**
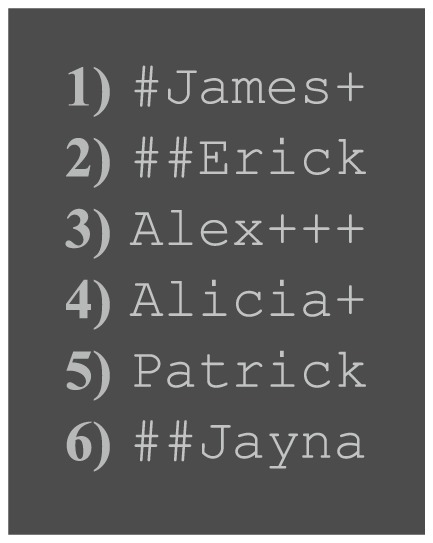
Example names. List of example names, formatted as stimuli. Note that name 3 would not be shown immediately after name 4 as they have 2 letters (‘A’ and ‘L’) in the same position.

We presented RSVP streams on a 20” LCD screen with a refresh rate of 60 Hz and a resolution of 1600×1200, placed at a distance of 60 cm from the participant. We used custom scripts that employed the Psychophysics toolbox version 3, running under Matlab 2010a. Stimuli were 16 point, light grey (75% white; RGB:190,190,190) monospaced, sans-serif characters presented on a dark (25% white; RGB:64,64,64) background. As a result, the visual angle for each stimulus was 0.48° in height and 2.48° in width, whereas the whole screen consisted of a rectangle of 28.52° by 37.56°. The Stimulus Onset Asynchrony (SOA) was 100ms. Each RSVP trial consisted of a stream of 15 items, plus a starting and finishing item. The starting item was XXXXXXX, presented for 800ms, in order to position participant’s focus on the stimulus presentation area. The finishing item was either ------- or  =  =  =  =  =  =  = , selected at random, and remaining on screen for 100ms. The response phase began by asking the participant to identify the finishing item. We used this to keep attention focused on the stream after the critical item (Probe, Fake or Irrelevant1/2) had been presented, thereby avoiding muscle artefacts caused by response preparation and initiation before stream end. Apart from starting and finishing items, all stimuli were common English proper names with a maximum length of 7 characters, and first letter capitalised. We padded shorter names using a randomising algorithm, with ‘#’ or ‘+’ characters blocked on each side of the word (Figure 1). Distractor names were chosen pseudorandomly: in order to avoid repetition, names could not contain two or more letters in the same position as their immediate predecessor. In addition, names which shared three or more letters in the same position as one of the critical items were not presented as distractors. We presented all stream items at the same screen location.

#### 2.1.2 Stimuli

As previously indicated, we call *Irrelevant1*, *Irrelevant2*, *Probe* or *Fake* stimuli *critical items*. These critical items could be the participant’s real name (*Probe*), their assumed name (*Fake*) or one of two preselected names, unknown to the participant (*Irrelevant1* or *Irrelevant2*). There were 3 blocks, each consisting of a random sequence of *Irrelevant1, Irrelevant2, Probe* and *Fake* trials. For each trial type, there were 50 RSVP trials. Each trial of 15 items contained only one critical item and 14 randomly chosen names as distractors. The position of the critical item within the stream was selected pseudorandomly, so that it had equal probability of appearing in the 5^th^ position (earliest) through to the 10^th^ position (latest).

We generated a set of possible names from the USA Social Security Administration database (http://www.ssa.gov/oact/babynames/). The 1000 top names from four different years (2009, 1969, 1929 and 1890) were combined into a single set of unique names. We only kept names shorter than 8 characters, resulting in a total set size of 3667 names. Prior to the start of the experiment, we presented participants with a subset of 12 possible female or male names, depending on their gender, from which they removed all names of people they knew well. Participants then chose one of the remaining names as their *Fake* name. After each RSVP stream, they were asked, on-screen, “did you see your name”? We had previously instructed participants to answer “Yes” if they had seen their Fake name and “No” otherwise, including when they saw their real name (the *Probe*) (participants’ responses to this question are reported in the supplementary material: Table S1 in Appendix S1). We chose two further names unfamiliar to the participant from the subset of twelve possible names and used them as *Irrelevant1* and *Irrelevant2*. Experimentally, we treated these identically; their only difference was in the (random) choice of name. Furthermore, Irrelevants were identical to distractors apart from the frequency with which they occurred over the course of the experiment (50 times each and approximately once per distractor).

#### 2.1.3 Experiments

In total 5 experiments (groups) were conducted, named as follows:

- *No countermeasures* (exp. 1)
- Countermeasure: *Probe as low salient* (exp. 2)
- Countermeasure: *Irrelevants as high salient 1* (exp. 3)
- Countermeasure: *Irrelevants as high salient 2* (exp. 4)
- *Innocents* (exp. 5)

The instructions described in the previous section were given to all participants (including those in the Innocents group). An additional set of instructions was given to participants assigned to each of the countermeasure groups (as described in Section 2.1.6 below). The first four experiments were conducted to assess the detection sensitivity of our method. In other words, their aim was to measure the hit rate. Similarly to our previous study [22], we evaluated the false alarm rate by conducting the “Innocents” experiment. Participants assigned to that group were not shown a Probe (i.e. their own first name). Rather, we replaced the Probe with an additional Irrelevant (*Irrelevant3)*, which was a name selected in the same fashion as the other two Irrelevants, and we assessed the probability of falsely ascribing guilt to the Innocent (this is discussed more in detail in Section 2.1.13). We estimated the area under the Receiver Operating Characteristic (ROC) curve (AUC) for the first four experiments by iterating through all possible alpha levels (from.0001 to 1). A hit rate (using p-values obtained in the given hit rate experiment) and a false alarm rate (based on the p-values obtained in the fifth experiment, the “Innocents”) were calculated for each alpha level setting. The resulting sensitivity and specificity ranges where used to estimate the AUC for the first four experiments.

#### 2.1.4 Participants

All participants were students or staff at the University of Kent. All were right handed. Participants were free from neurological disorders and had normal or corrected-to-normal vision. Only native English speakers participated in the experiment. The study was advertised publicly and all were paid 8 pounds (GBP) for participating. Details for each group are as follows:

- No countermeasures (exp. 1): 12 participants, 5 male, 7 female. Age range: 21–27 (*M:* 20.7, *SD:* 2.4).
- Probe as low salient (exp. 2): 10 participants, 5 male, 5 female. Age range: 19–23 (*M:* 19.9, *SD:* 1.2).
- Irrelevants as high salient 1 (exp. 3): 10 participants, 4 male, 6 female. Age range: 19–33 (*M:* 21.7, *SD:* 4.1).
- Irrelevants as high salient 2 (exp. 4): 10 participants, 5 male, 5 female. Age range: 18–29 (*M:* 21.1, *SD:* 3.4).
- Innocents (exp. 5): 8 participants, 4 male, 4 female. Age range: 19–20. (*M:* 18.9, *SD:* 0.8).

An additional participant took part in the Probe as low salient experiment, but was excluded from analysis due to a high number of artefacts (our inclusion threshold was at least 20 valid trials in all conditions). All participants took part in only one experiment.

#### 2.1.5 Ethics

This study was approved by the University of Kent Psychology Ethics Committee, which follows the guidelines set by the British Psychological Society regarding experiments with human participants. The study was approved as reference number 20101504. Written consent was obtained from all participants.

#### 2.1.6 Countermeasures

Participants who were assigned to one of the countermeasure groups were given an additional set of instructions. These instructions were based on the type of countermeasure being applied:

Probe as low salient (exp. 2): participants were told that the detector works by detecting when they see their real name. In order to avoid this, they were told to concentrate hard on “not seeing their real name”.

Irrelevants as high salient 1 (exp. 3): participants were told that two further names appear frequently in the experiment (i.e. the Irrelevants). They were told that their task was to count the number of times that each occurs in the experiment.

Irrelevants as high salient 2 (exp. 4): participants were instructed that two further names (the Irrelevants) appear in the experiment. In this case, their task was to identify at least one of them and, once identified, pretend that it was their real name (although they were still instructed to answer ‘No’ to the ‘Did you see your name?’ question, even after identifying one of the Irrelevants).

Participants were briefed verbally before the start of the experiments: they were informed of how the deception detector works prior to the start of EEG recording. They were instructed to perform only one countermeasure strategy, depending on the group they were assigned to. In addition to the verbal briefing, they were given written instructions. The exact written instructions that were given to participants can be found in the supplementary material, Appendix S2.

#### 2.1.7 Recording Apparatus

We recorded data using a Brain Products QuickAmp recorder (BrainProducts, Munich, Germany). We bandpass filtered data at recording, with a low-pass of 85 Hz and a high-pass of 0.30 Hz. We recorded Electroencephalographic data from the Fz, Cz, P3, Pz, P4, A1 and A2 electrodes using the standard 10–20 system (Jasper, 1958). We recorded electrooculograms from the left and right eyes using two bipolar HEOG and VEOG electrodes. During recording, we used the average of all channels as reference (common reference). We kept impedances below 5 kOhm.

#### 2.1.8 Analysis Procedure

We analysed data with EEGLAB version 9 under Matlab 2010a [26] and custom scripts which implemented the methods described in the following sections. At analysis, we software filtered data with a low-pass of 45 Hz and high-pass of 0.5 Hz. We applied a notch filter between 8 Hz and 12 Hz to remove ssVEP oscillations set-up by the RSVP stream. We re-referenced data to the average of the combined mastoids (electrodes A1 and A2). We detected eye blinks by marking any activity below –200 µV or above +200 µV in the EOG channels as artifactual. Additionally, trials were automatically inspected so that any trial containing electrical activity below –50 µV or above +50 µV in the remaining EEG channels was rejected. Both these procedures only considered data ranging from –500ms to 1000ms from critical item (Probe, Fake, Irrelevant1/2) onset. The number of trials remaining after artefact rejection, per condition, ranged between 23 and 50 (*M*: 46.35, *SD*: 4.34) across all experiments. We calculated ERPs, on which the following analysis is based, using –100ms to 1000ms stimulus-locked windows, baseline corrected from –100ms to 0ms.

#### 2.1.9 P3 differences

For each condition (Probe, Fake and Irrelevant2), we estimate three different P3 measures, named P3b-Pz, P3a-Fz and P3a-Cz. This is done on a participant-by-participant basis (on participant-level ERPs). These three measurements are determined from the point-wise difference between the ERP of the given condition and the ERP of the Irrelevant1 condition, which plays the role of baseline. The measure employed is the peak-to-peak value of the difference wave (condition minus Irrelevant1). In more detail, initially, the raw difference between the ERP of the given condition and the ERP of the Irrelevant1 condition is calculated. The result of this operation is a difference wave, which in certain conditions contains a P3 signal. In order to determine the intensity of the signal, a peak-to-peak measurement procedure is applied to this difference wave. Two parameters of this procedure vary depending upon the channel: P3b parameters are applied at Pz, P3a parameters at Fz and Cz. The first parameter is the start of the time window in which we *search* for the P3 (strictly, search for its highest and lowest peaks), we call this the *bounding* P3 window. For the P3b, the bounding window starts at 300ms from critical item onset and ends at 1000ms from critical item onset, whereas for the P3a the bounding window starts at 150ms and ends at 1000ms (the search for the positive peak of the P3a was limited, though, to between 150ms and 300ms from critical item onset, as detailed in the next section). We consider the extent and placement of these bounding windows to be a priori justified by the P3 literature and also directly inherited from [22], and thus not subject to multiple comparison’s correction [27]. (Note, preserving window placement and analysis parameters exactly from a previous study is the most certain way to guard against statistical bias, i.e. inflation of Type I errors, since the probability that the “background” noise variability in the data has a similar pattern across the two studies is very small.) As just discussed, the second parameter that varies between P3b and P3a analysis is the presence of a boundary that limits the search for the highest peak, which is present only for the P3a analysis (this is discussed in more detail in the next section).

#### 2.1.10 Peak-to-Peak

The peak-to-peak procedure we applied to the difference waves (generated following the procedure described in the preceding section), determines the disparity between the highest peak and the following lowest (typically negative) peak in the specified P3 bounding window. Note that peaks here are not identified based on single time points, but rather on averages computed across relatively small windows of time points. This usage is consistent with peak-to-peak measurements used in previous P3 deception detection research [17]. (For the purpose of this paper, the word peak will always refer to such an average). Hence, peaks were identified as the highest or lowest averages across inner windows of 100ms, i.e. each peak corresponds to the mean voltage of that window. (We use the term *inner* window to refer to a time interval across which we calculate the average amplitude.) The procedure finds the highest peak first, by iterating through all 100ms (inner window) intervals from the start of the P3 bounding window until its end. In other words, we slide a 100ms interval across the bounding window, looking for the interval with the highest average. For the P3a, the search for the highest peak ends at 300ms from critical item onset. The presence of this boundary prevents the P3b (whose start was previously pinpointed at 300ms in RSVP experiments [24]) from being detected as the highest peak of the P3a. Note that the end of the P3a bounding window coincides with the end of the P3b bounding window. This is because the variability in the latency of the P3a negative bounce-back is too high to allow for an alternative placement. Consequently, we decided to place a broad window to avoid the risk of over-fitting search windows to our ERP patterns. This approach is statistically safe, as we demonstrated in the “Intrinsic validity test” presented in our previous study [22]. This is because the same broadness of search window is applied under the null hypothesis, i.e. under our randomisation.

After the highest peak is found, the procedure then continues iterating from the first non-overlapping position that followed the highest peak until the end of the P3 bounding window, searching for the lowest peak. The peak-to-peak measurement is finally calculated as highest minus lowest.

Subtracting, in this way, lowest from highest peak in the P3 bounding window, will, in most cases, yield a positive peak-to-peak value. Thus, in our group-level P3 analysis, a comparison against zero is inappropriate, and we require a ‘no-effect’ baseline to compare against. The inclusion of Irrelevant2 trials gives this baseline. Thus, we also calculate an Irrelevant2 peak-to-peak by, in the same way, subtracting out the Irrelevant1 ERP and calculating an Irrelevant2 peak-to-peak value on the Irrelevant2 minus Irrelevant1 difference wave. We compare the Probe peak-to-peak values to the Irrelevant2 peak-to-peak values across participants using a t-test. Raw peak-to-peak data for all participants are provided in the supplementary material (Table S2 in Appendix S1).

#### 2.1.11 First Level: Single dimension randomisation

For each electrode, we undertake a separate first level randomisation; thus, electrodes Fz, Cz and Pz serve as single dimensions. We then perform a second level analysis, which determines a combined significance across these dimensions/electrodes. We discuss these first level randomisations here.

We applied a randomisation procedure in order to determine a participant’s null hypothesis distribution. (Note, a trial is effectively a triple, with P3a-Fz, P3a-Cz and P3b-Pz segments. In this way, we maintain the correlations across electrodes within trials.) Before the procedure started, the least number of valid trials between the Probe and Irrelevant1 conditions was determined (valid trials are free of eye blinks and other artefacts; Section 2.1.8 detailed our artefact rejection procedure, including the typical number of trials rejected in this study); we call this number *m*. *m* trials were, then, selected from the Probe condition, and *m* from the Irrelevant1 condition. These selections were performed at random, without replacement.

The randomisation procedure was the same at each electrode (Pz, Fz, Cz); for each it proceeded as follows. First, two vectors (each of size *m*) were randomly populated with the 2 × *m* selected trials. Note, under the null hypothesis, Irrelevant1 and Probe trials would be samples from the same distribution - the null distribution - and would thus be exchangeable. Second, a pair of ERPs were generated, one from each vector. One of these ERPs notionally playing the Probe role and the other the Irrelevant1 role. A peak-to-peak difference between the two ERPs was then calculated. The procedure repeated until 10,000 values were obtained; these 10,000 correspond to the null hypothesis distribution.

A p-value was determined as follows: the true observed value was obtained from the (true) ERPs of the given participant, as the peak-to-peak of the difference between the (true) Probe and (true) Irrelevant1 conditions. Since, as previously discussed, we apply this same procedure at the three electrodes (Pz, Fz, Cz), we obtain three, Probe against Irrelevant, p-values.

#### 2.1.12 Second Level: Combined analysis

For each participant, the data from the three single dimension randomisations (P3a-Fz, P3a-Cz and P3b-Pz) described the previous section were used to compute a joint p-value under a Fisher combined probability test. A number of methods for combining different dimensions of statistical significance have been considered [28], [29]. The Fisher method (discussed in Hayasaka and Nichols) treats the different dimensions consistently, since by combining *p-values* of individual dimensions, it automatically normalises into a common comparable measure. A dimension where there are very large (raw) differences between data points would have a disproportionate effect on the combined significance without such normalisation.

To determine a combined p-value for one participant across electrodes (P3a-Cz, P3a-Fz and P3b-Pz), we first calculated 10,000 single dimension p-values, for each electrode. Each such p-value reflects where one data point (denoted *d*), arising from our original random resampling (which was described in Section 2.1.11), sits in its single dimension randomisation distribution. That is, a p-value was obtained by determining the proportion of the 10,000 values present in the single dimension randomisation distribution that were above *d*. This gave us 30,000 p-values: 10,000 for each electrode/dimension, with associations across dimensions, such that data point *i* in the P3a-Fz electrode corresponds to point *i* in the P3a-Cz electrode and point *i* in the P3b electrode (since these three data points were generated from the same random sample). Finally, 10,000 Fisher scores were obtained by using the following formula:
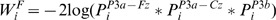



where, *i* ranges over the 10,000 random samples. The key aspect of this formula is that the p-values from single dimensions are multiplied.

Similarly, a Fisher score was calculated on the true observed data point using the same formula. An overall, cross dimension p-value was, then, obtained by calculating how many of the 10,000 random sample Fisher scores were above the true observed Fisher score, and then dividing by 10,000. When calculating Fisher scores, values of p = 0 (which would result in the formula returning infinity) were replaced by the smallest legitimate p-value, 0.0001 (1/10,000). Further discussion of our implementation of Fisher’s combinatorial method, including a sanity check regarding its “intrinsic” false positive rate, can be found in our previously published paper [22].

It should be noted that there has been debate concerning the appropriateness of Fisher combining in meta-analyses. Specifically, it has been argued that the Fisher method incurs a loss of precision. [30] is a good discussion of these issues. Importantly, with simulated data, [30] showed that there was no inflation of the type one error with Fisher combining; that is, when the null hypothesis is true (i.e. on pure noise data) the probability of obtaining a significant result is the alpha level. But, there was a loss of precision when data sets containing an effect were considered. The Type I error rate, though, is the most fundamental criterion for judging the validity of a method, i.e. that the false positive rate is not inflated, and, indeed, as just stated, we provide such a demonstration in [22], i.e. that in the context of our experiments on EEG data, there is no type one error inflation.

It is though certainly not optimal that Fisher combining does not accurately combine probabilities in non-null meta-analysis data sets. This though is not in fact relevant to our use of the Fisher method. This is for two reasons. Firstly, Whitlock considers the Fisher in the context of a Chi-squared test – we do not perform such a test, rather our permutation procedure is nonparametric and, thus, does not make any assumptions about distribution shape. Secondly, and most importantly, we are not performing a meta-analysis. Whitlock’s demonstration of imprecision is in the context that the true probability for the tests being combined is the same – as it would be if each experiment was, at least theoretically, a replication of the others – this is the meta-analysis case. In contrast, we are combining p-values from three electrodes – these are not identical tests. For example, the P3a component seen at Fz is very different to the P3b at Pz. Thus, Whitlock’s assessment of precision against a single “true” p-value does not apply and, indeed, in our context there is no “ground-truth” overall p-value to assess our Fisher combining against.

In this context, the critical criteria for judging a statistical method’s validity are, first, the Type I error rate and, second, that the method has sufficient statistical power to give significant results when non trivial effects are present. The first of these is justified by our intrinsic false positive test in [22] and the second, informally, from our success in [22] in detecting identity deception.

#### 2.1.13 False Positive Rate

In one respect, the randomisation procedure controls the false positive rate, by explicitly calculating the null hypothesis distribution and deriving a p-value from it; that is, by considering the consequence of interpreting the Probe and Irrelevant as samples from the same distribution. However, the true *empirical* false positive rate is the chance of interpreting a non-deceiving participant as deceiving and that requires considering a situation in which what the experimenter considers to be a Probe in fact really is an Irrelevant. Put another way, our randomisation procedure calculates the false positive rate when the Probe is *hypothetically* treated as an irrelevant, but, because all participants are lying about their identity in our main experiment, the Probe was in fact indeed their real name. But, there remains the possibility that participants behave differently if there really is no condition in which their name is present. For example, it might be that without a Probe to notice, Irrelevants would be more easily seen. This is the question we explore in our empirical false positive rate experiment.

Specifically, we ran our experiment on the “Innocents” group (experiment 5, as per Section 2.1.4). We utilised exactly the same stimulus presentation, stimuli, recording apparatus and technique previously highlighted. The only difference being that there was no Probe, but rather three Irrelevants: Irrelevant1, Irrelevant2 and Irrelevant3, each selected at random from the set of possible names, without informing the participant of their identity. Thus, their real name did not appear frequently in the experiment (it could only appear as a distractor: the chance of this happening in any given trial was 0.38%, i.e. less than half a percent). Handling of the Fake was unchanged.

This gave us three identical conditions for each participant: Irrelevant1, Irrelevant2 and Irrelevant3, each of which comprised three sets of trials - one for each electrode: Fz, Cz and Pz. The three Irrelevants at each electrode yielded six pairwise comparisons, since there are six permutations of three, e.g. (Irrelevant1, Irrelevant2), (Irrelevant1, Irrelevant3), (Irrelevant2, Irrelevant3), (Irrelevant2, Irrelevant1), etc. We ran our statistical analysis on each such pair, with the first in the pair playing the (notional) Probe role and the second the Irrelevant role. Across the eight participants, this gave us 48 data sets, each comprising notional Probe at Fz, Cz and Pz and Irrelevant at Fz, Cz and Pz. We analysed each data set with single dimension randomisations for Fz, Cz and Pz and then a Fisher combining. This gave us 48 tests of an empirically-enforced null hypothesis. From this we can determine an approximate false positive rate.

### 2.2 Questionnaires

After the RSVP phase of each experiment, we explored participants’ memory for presented names, by administering two questionnaires to every participant. The first was called *Recall*, which was followed by a second, called *Recognition*. In the Recall test, participants were asked to write five names that they thought appeared often during the experiment (including the Fake and Probe). On the Recognition questionnaire we wrote five names and asked each participant to give their confidence that any of them appeared. These ratings ranged from 1 (lowest confidence/name did not appear) to 5 (highest confidence/name appeared very often). These five names were the Probe, Fake, Irrelevant1, Irrelevant2 and *Noncritical*. The Noncritical was selected from the unfamiliar names in the initial list of 12 names (which was presented to each participant during prescreening before the start of the experiment). Unlike the other names present in the Recognition questionnaire, the Noncritical did not appear often in the experiment: it could only appear as a distractor. The probability of the Noncritical appearing in a trial was 0.38%: the same as any of the possible 3,663 distractor names. Here, recall, which was deliberately performed first, is the test most relevant to the feasibility of the Irrelevants as high-salient countermeasure. In particular, in a deployed deception detector, suspects would not be cued with the identities of potential Irrelevants (including the real ones) during lie detection, as they are in a recognition test.

Recall test results were analysed by computing a contingency table for each countermeasure strategy. Statistical analysis on the contingency tables was performed using Fisher’s exact test, normally used when the number of samples is very small [31]. We took the No countermeasures experiment (exp. 1) as baseline and in each case assessed whether the particular countermeasure employed in experiments 2, 3 or 4 changed performance on the relevant critical item: Probe in exp. 2 and Irrelevant in exps. 3 and 4. Specifically, in the first test, we compared the amount of times that the Probe was recalled between the No countermeasures experiment (exp. 1) and the Probe as low salient experiment (exp. 2). This was a left tailed test and indicated the probability that the Probe was not recalled less often in the Probe as low salient experiment. The remaining four recall tests compared the amount of times that Irrelevants were recalled in the No countermeasures experiment (exp. 1) to the number of times they were recalled in the Irrelevants as high salient experiments (exps. 3 and 4). The tests were four since we compare each Irrelevant (1/2) separately, for each pair of experiments (exp. 1 against exp. 3 and exp. 1 against exp. 4). We performed right tailed comparisons, since we were interested in assessing an increase of recall in the Irrelevant conditions, when countermeasures are applied.

We analysed the responses given in the recognition questionnaire using a Wilcoxon rank-sum test (also called Mann–Whitney *U*). This test is considered to be appropriate when comparing ordinal and non normally distributed variables such as those obtained via our recognition questionnaire [32]. The test compared the scores given by each participant for the Noncritical against the scores assigned to each “Critical” item (Fake, Probe, Irrelevant1 and Irrelevant2). Here, responses to the Noncritical serve as baseline, that is, reflect the bias in confidence responses, i.e. to an item not appearing frequently in the experiment. The test was left tailed (i.e. the alternative hypothesis was that the Noncriticals obtained smaller scores than the Criticals). A test was performed for each experiment. The results of these analyses are reported in Section 3.2.3.

## Results

### 3.1 Group-Level Analysis

**Figure 2:**
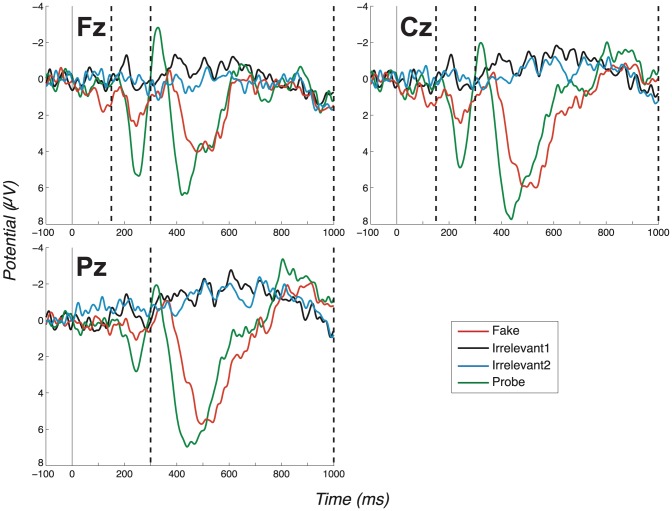
Grand average ERPs for the “No countermeasures” experiment (exp. 1), all channels. (Positive plotted down.) Vertical dashed lines mark the regions in which we search for P3 peaks. The search window for the positive peak of the P3a ends at 300ms, which coincides with the start of the window for the P3b at Pz (as described in Section 2.1.9, we search for the positive peak of the P3a between 150–300ms at Fz and Cz, while for the P3b we start at 300ms, at Pz. All bounding windows end at 1000ms from critical item onset). Note the large P3a for the Probe condition, which is much less pronounced in the Fake condition. Also note the large P3b for the Probe condition (Pz channel).

There are, in fact, two ERP components that in all four of our experiments, enable us to distinguish Probe from Irrelevant: a fronto-central complex, which we interpret as a P3a, and a parietal complex, which we interpret as a P3b. Our first experiment demonstrated clearly distinct group-level (Fz, Cz & Pz) grand average ERP profiles for Probes compared to Irrelevants in a basic, no-countermeasures, condition, see Figure 2. The size of this effect was reflected in a highly significant group-level difference between Probe and Irrelevant (P3a-Fz: *t*(11)  =  5.57, *p*<.0002; *d*  =  1.61. P3a-Cz: *t*(11)  =  5.72, *p*<.0001; *d*  =  1.65. P3b-Pz: *t*(11)  =  5.91, *p*  = .0001; *d*  =  1.70).

**Figure 3:**
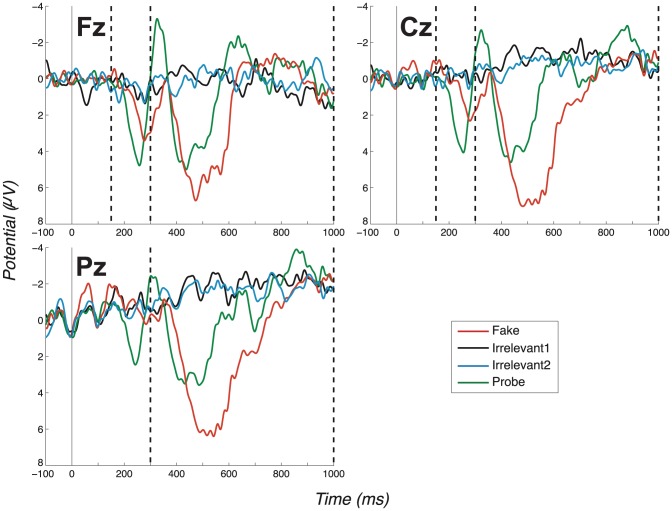
Grand average ERPs for the “Probe as low salient” experiment (exp. 2), all channels. (Positive plotted down.) Vertical dashed lines mark the regions in which we search for P3 peaks. Again, the search window for the positive peak of the P3a ends at 300ms, coinciding with the start of the P3b-Pz window. Note the P3a for the Probe condition is identifiable as a large distance between positive and negative peaks. Also note the large P3b for the Probe condition (Pz channel).

In the second experiment, we specifically tested the Probes as low-salient countermeasure. The procedure was unchanged from experiment 1, apart from extra pre-experiment instructions. Specifically, we told participants how the identity detector worked, i.e. that it detects the brain state generated when one’s own name is seen, and to concentrate hard on “not seeing their real name”. This Probe as low-salient countermeasure failed to remove our ERP effect. Specifically, group-level grand average ERPs, see Figure 3, again exhibited clear and highly significant differences between Probe and Irrelevant (P3a-Fz: *t*(9)  =  6.07, *p*<.0002; *d*  =  1.92. P3a-Cz: *t*(9)  =  4.83, *p*  = .0009; *d*  =  1.53. P3b-Pz: *t*(9)  =  4.06, *p*  = .0029; *d*  =  1.28).

**Figure 4:**
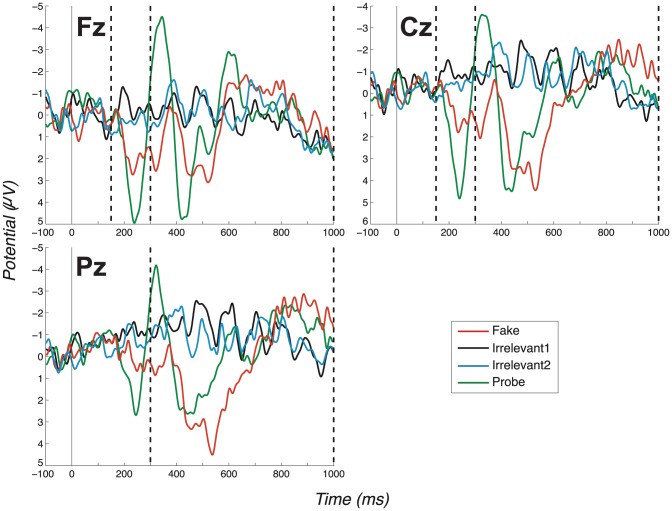
Grand average ERPs for the “Irrelevant as high salient 1” experiment (exp. 3), all channels. (Positive plotted down.) Vertical dashed lines mark the regions in which we search for P3 peaks. Again, the search window for the positive peak of the P3a ends at 300ms, coinciding with the start of the P3b-Pz window. Note the large P3a for the Probe condition, identifiable as a large distance between the positive and negative peaks.

In the third experiment, we tested the Irrelevants as high-salient countermeasure. The procedure was, again, unchanged from experiment 1, apart from added pre-experiment instructions. Specifically, we told participants that two unidentified names (i.e. the Irrelevants) would occur frequently, to attempt to ‘see’ these names on the basis of their frequent presentation and accordingly, to count how often each occurred. The group-level (grand average) ERPs arising from this manipulation, see Figure 4, again exhibited a clear difference between Probe and Irrelevant, which remained significant (P3a-Fz: *t*(9)  =  5.82, *p*  = .0003; *d*  =  1.84. P3a-Cz: *t*(9)  =  3.74, *p*  = .0046; *d*  =  1.18. P3b-Pz: *t*(9)  =  4.85, *p*  = .0009; *d*  =  1.53).

**Figure 5:**
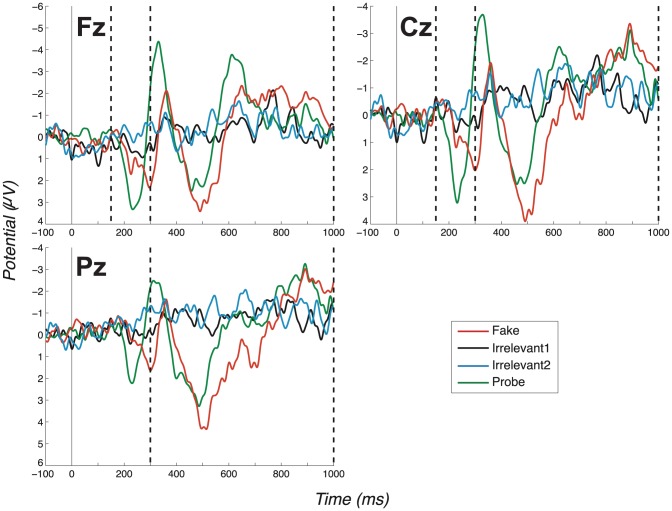
Grand average ERPs for the “Irrelevant as high salient 2” experiment (exp. 4), all channels. (Positive plotted down.) Vertical dashed lines mark the regions in which we search for the P3. Again, the search window for the positive peak of the P3a ends at 300ms, coinciding with the start of the P3b-Pz window. The Probe shows both a P3a and a P3b (Pz channel).

Experiment three leaves the possibility that Irrelevants might not have generated a P3-pattern similar to the Probe, because the task performed to the Irrelevant (when seen) induced a task set different to that applied to Probe or, indeed, Fake. Thus, experiment four was identical to experiment three, apart from an instruction change. This required that, once ‘seen’, rather than counting occurrences of Irrelevants, participants would ‘pretend’ it was their real name. In this way, they would treat Irrelevants as similarly as possible to how they treat Probes (they cannot, of course, treat them identically, since they are not their true name). As anticipated, the instruction change did not substantially alter the ERP pattern. Consequently, group-level grand average ERPs, see Figure 5, again showed a clear difference between Probe and Irrelevant, which remained highly significant (P3a-Fz: *t*(9)  =  5.51, *p*  = .0004; *d*  =  1.74. P3a-Cz: *t*(9)  =  6.16, *p*  = .0002; *d*  =  1.95. P3b-Pz: *t*(9)  =  4.85, *p*  = .0009; *d*  =  1.53). All t-test results are aggregated in Table 1, where we also consider Bonferroni correction for multiple comparisons.

**Table 1:** Group level t-test results for all experiments and channels (Probe against Irrelevant2).

Group	Channel	Outcome	Confidence interval (µV)
**No C/M**	P3a-Fz	*t*(11)* = *5.57, *p = *.0002; *d = *1.61	3.0884 ∼ 7.1197
**(exp. 1)**	P3a-Cz	*t*(11)* = *5.72, *p = *.0001; *d = *1.65	2.9892 ∼ 6.7319
	P3b-Pz	*t*(11)* = *5.91, *p = *.0001; *d = *1.70	5.9681 ∼ 13.0565
**Probe Low**	P3a-Fz	*t*(9)* = *6.07, *p = *.0002; *d = *1.92	2.7534 ∼ 6.0243
**(exp. 2)**	P3a-Cz	*t*(9)* = *4.83, *p = *.0009; *d = *1.53	2.5583 ∼ 7.0705
	P3b-Pz	*t*(9)* = *4.06, *p = *.0029; *d = *1.28	2.5068 ∼ 8.8257
**Irr High 1**	P3a-Fz	*t*(9)* = *5.82, *p = *.0003; *d = *1.84	2.5129 ∼ 5.7096
**(exp. 3)**	P3a-Cz	*t*(9)* = *3.74, *p = *.0046; *d = *1.18 *	1.2428 ∼ 5.0401
	P3b-Pz	*t*(9)* = *4.85, *p = *.0009; *d = *1.53	2.6689 ∼ 7.3424
**Irr High 2**	P3a-Fz	*t*(9)* = *5.51, *p = *.0004; *d = *1.74	2.2431 ∼ 5.3690
**(exp. 4)**	P3a-Cz	*t*(9)* = *6.16, *p = *.0002; *d = *1.95	1.9261 ∼ 4.1609
	P3b-Pz	*t*(9)* = *4.85, *p = *.0009; *d = *1.53	1.9618 ∼ 5.3868

* By applying Bonferroni correction for 12 comparisons, we obtain a significance threshold of *p* = .0042. This results in one (marginal) failure to find a significant difference between Probe and Irrelevant2 on single channel data.

In addition, participants were behaving as instructed, since they responded “yes” to the (end of stream) “Did you see your name?” question very often for Fake and very infrequently for both Probe and Irrelevants. We verified this pattern using a Wilcoxon rank-sum test, comparing the number of “yes” responses between conditions and experiments (see Tables 2 and 3 for details).

**Table 2:** Wilcoxon rank sum test on the number of times that “Yes” was answered to the “Did you see your name?” question, between the No countermeasures experiment (exp. 1) and all other experiments.

Experiment	Fake	Probe	Irrelevant1	Irrelevant2
**Probe Low (exp. 2)**	0.7639	0.6259	0.0366	0.3531
**Irr High 1 (exp. 3)**	0.0834	0.6331	0.0428	0.0333
**Irr High 2 (exp. 4)**	0.6667	0.7836	0.3189	0.0464
**Innocents (exp. 5)**	0.8461	0.9040	**0.0005** *	0.0294

* Significant results after Bonferroni correction (16 comparisons) are shown in bold.

*Note.* The performed test was a two-tailed test. This table shows that, in general, participants were answering “Yes” to the “Did you see your name?” question similarly between the No Countermeasure experiment and the other experiments in most cases. Only one significant result is present for the Irrelevant1 condition in the Innocent experiment. This is due to a few participants misinterpreting the instructions in the No Countermeasure condition, who answered “Yes” in trials in which they did not see their real name (the Probe), including Irrelevant trials. This is detailed in the supplementary material (Table S1 in Appendix S1).

**Table 3:** Wilcoxon rank sum test on the number of times that “Yes” was answered to the “Did you see your name?” question, comparing between the Fake and the other conditions/experiments.

Experiment	Probe	Irrelevant1	Irrelevant2
**No Countermeasures (exp. 1)**	<0.0001	0.0004	0.0002
**Probe Low (exp. 2)**	0.0001	0.0001	0.0001
**Irr High 1 (exp. 3)**	0.0001	0.0001	0.0001
**Irr High 2 (exp. 4)**	0.0001	0.0002	0.0002
**Innocents (exp. 5)**	0.0001	0.0001	0.0001

*Note.* Right tailed test, all significant, showing that participants were following instructions correctly, answering "Yes" more often after Fake trials in all experiments (Bonferroni correction for 15 comparisons puts the significance threshold at p = 0.0033). When aggregating across all experiments, we get *p*<.0001 for all conditions.

#### 3.1.1 Early fronto-central complex

Now, considering all experiments, we observe a clear fronto-central full oscillation cycle, which is large and early for the Probe, medium-sized and slightly later for the Fake and absent for Irrelevant1 and Irrelevant2, as shown in the grand averages for Fz and Cz (Figures 2–5). This component is initially positive, with a following damped negative deflection. As in our previous work [22], we call this a P3a (we return to the reasoning behind this naming in the discussion, Section 4.2). Our key group-level P3a statistical test is a paired t-test of a peak-to-peak analysis of Probe P3a and Irrelevant2 P3a across participants. We performed a test for each experiment. The outcome of these tests are reported in Table 1, along with results for the Cz and Pz channels (the individual peak-to-peak values on which the t-tests were computed are provided in the supplementary material: Table S2 in Appendix S1). All paired t-tests were highly significant, except for one marginally significant outcome (after Bonferroni correction).

#### 3.1.2 P3b component

**Figure 6:**
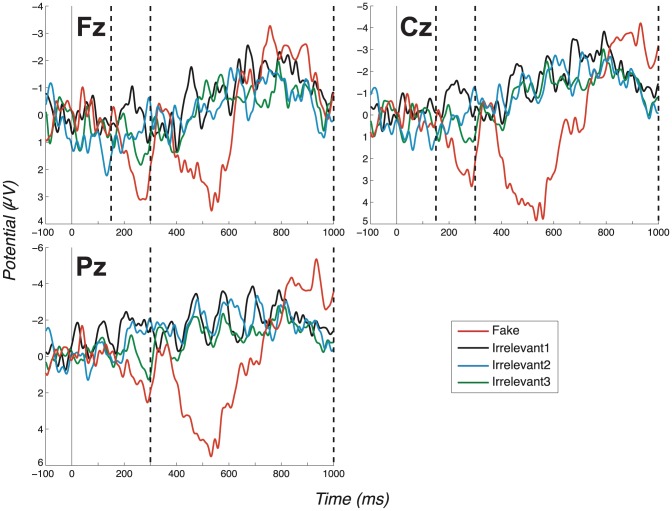
Grand average ERPs for the *Innocents* (exp. 5). (Positive plotted down.) Note the absence of P3a (Fz and Cz) and P3b (Pz) for all conditions but the Fake.

Figures 2–5 present grand averages for all experiments. In the Pz channels, positive deflections in the identified P3b region are clearly evident for Fake and Probe. The P3b elicited by the Fake often (but not always) has the largest amplitude. The Probe also generates a robust group-level P3b, which is somewhat smaller and earlier than the Fake P3b. As for the P3a analysis, peak-to-peak P3b values for both Probe and Irrelevant2 were compared and are provided in Table S2 in Appendix S1. Results of all t-tests of Probe against Irrelevant, which resulted in very significant differences between the two conditions, are summarised in Table 1. As expected, the only P3b pattern that arises from the Innocents grand average was obtained from Fake trials (Figure 6).

### 3.2 Analysis by Individual

While a strong group-level effect is indicative, the true test of a deception detector is at the individual-level; that is, individuals need to be demonstrated to be deceiving. Accordingly, using a Monte Carlo permutation test, see Section 2.1.11 for details, we were able to show that, out of the 12 participants in experiment one (No countermeasures), 10 differentially processed the Probe on the P3a-Fz channel, 9 on P3a-Cz and 11 on the P3b-Pz (see Table S3 in Appendix S1 for p-values calculated on individual electrodes for all participants on all experiments). Furthermore, using Fisher combining to aggregate across the three P3 measures, all 12 participants had a Probe pattern distinct from Irrelevant, see Table 4. The AUC for this experiment was 0.9983. These findings demonstrate the base effectiveness of the Fringe/P3-Rapid method when no countermeasures are applied.

**Table 4:** Result summary table from per-individual combined (i.e. across three dimensions) randomisation analysis, all experiments.

Outcome (p-values)			
No C/M	Probe Low	Irr High 1	Irr High 2
(exp. 1)	(exp. 2)	(exp. 3)	(exp. 4)
0.0008	<0.0001	0.0302	0.0057
<0.0001	<0.0001	0.4919*	0.0007
<0.0001	0.0761*	0.0001	<0.0001
0.0001	0.0004	0.0002	0.0048
<0.0001	0.0560*	0.0006	0.0002
0.0009	<0.0001	<0.0001	<0.0001
<0.0001	0.0017	<0.0001	0.0037
<0.0001	0.0324	<0.0001	0.0181
0.0049	0.0001	0.0005	0.0397
0.0006	<0.0001	<0.0001	0.0070
0.0001			
0.0243			

* Participants whose p-value was above 0.05 are indicated with an asterisk (and in bold if p > 0.1). Three misses out of 42 are present, two of which are marginal. This is an extremely small Type II error rate. Note that rows are not meant to identify specific participants (this would imply that most took part in all experiments). For example, row 4 in this table would involve four participants, each of which was the fourth participant in an experiment.

Per-individual analyses in the second experiment (Probe as low salient) were again effective, with Probe significantly different to Irrelevants for 8/10 participants at Fz, 9/10 at Cz and 6/10 at Pz, and 8/10 distinguished under (Fisher) combined analysis, with the two misses being only marginal, see Table 4. Thus, we found no evidence that volitional control can direct subliminal search ‘not to see’ a highly salient stimulus sufficiently to confound our deception detector. The resulting AUC was 0.9854.

Irrelevant as high salient 1, the third experiment, demonstrated a strong effect at the individual level, with Probe significantly different to Irrelevant for 8/10 participants at Fz, 8/10 at Cz and 8/10 at Pz, and 9/10 under (Fisher) combined analysis, see Table 4. For this method, we calculated an AUC of 0.95. Thus, the effect of interest was not reduced sufficiently to suggest volitional control is able to direct the subliminal search system to ‘see’ Irrelevants effectively enough to confound our ERP analysis.

Analysis of the fourth experiment was, once again, effective, with Probe significantly different to Irrelevant for 6/10 participants at Fz, 9/10 at Cz and 8/10 at Pz. Most importantly, 10/10 participants were distinguished under (Fisher) combined analysis, see Table 4. The resulting AUC was 0.9938. Thus, the findings here and in experiment three are similar, suggesting that volitional control is not able to direct subliminal search to unknown frequent names sufficiently to modulate the ERP signatures that underlie our case.

**Figure 7:**
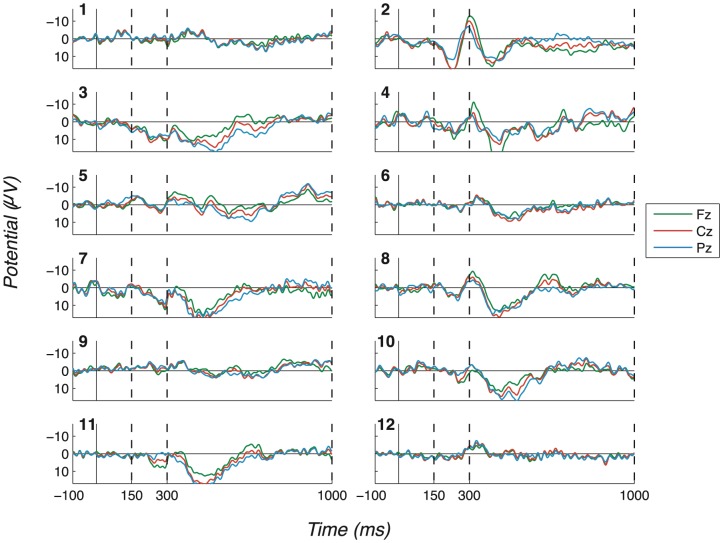
Difference waves for the “No countermeasures” experiment (exp. 1), all channels and participants. (Positive plotted down.) Probe - Irrelevant1 ERP difference waves, for each participant, are shown in this figure. Dashed vertical lines mark the start and end of P3 bounding windows: 150–300ms for P3a positive peak and 300–1000ms for P3b. Clear oscillations can be seen, suggesting that Probe stimuli were clearly perceived by participants, unlike Irrelevant1 stimuli.

**Figure 8:**
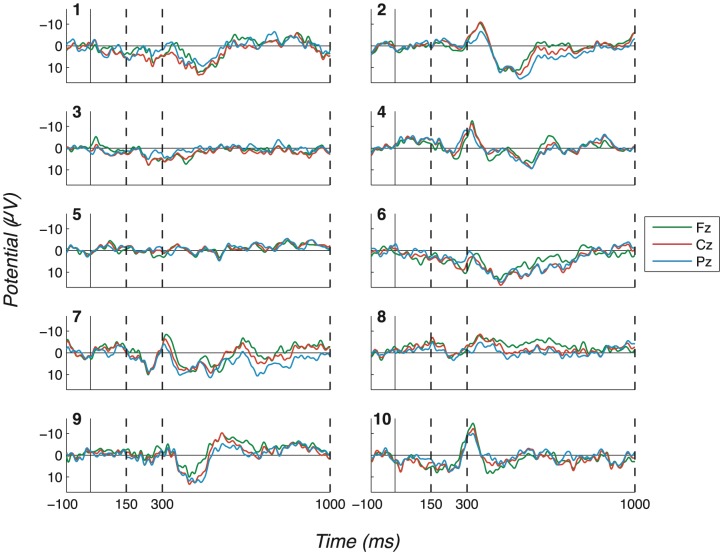
Difference waves for the “Probe as low salient” experiment (exp. 2), all channels and participants. (Positive plotted down.) Like Figure 7, this figure displays Probe - Irrelevant1 difference waves, in this case for the “Probe as low salient” experiment. This figure does not differ much from Figure 7, as strong P3 responses were elicited in most cases. Dashed vertical lines mark the start and end of P3 bounding windows: 150–300ms for P3a positive peak and 300–1000ms for P3b.

**Figure 9:**
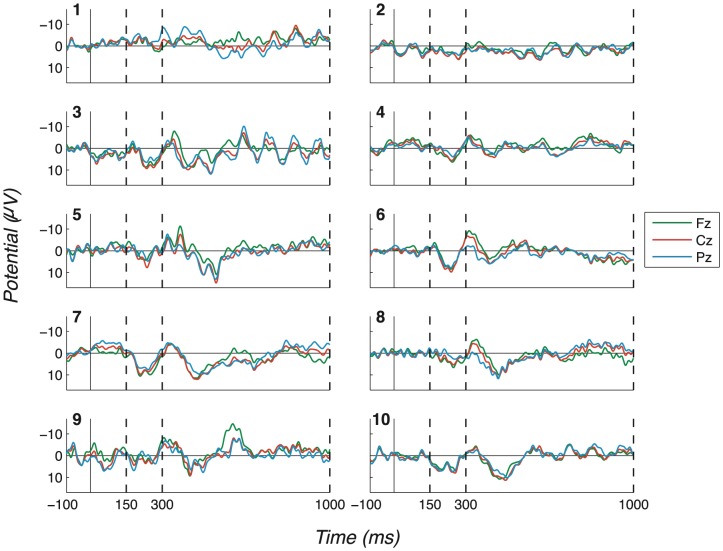
Difference waves for the “Irrelevant as high salient 1” experiment (exp. 3), all channels and participants. (Positive plotted down.) Similarly to Figures 7 and 8, this figure displays Probe - Irrelevant1 difference waves, in this case for the “Irrelevant as high salient 1” experiment. This figure does not differ much from Figure 7, as strong P3 responses were elicited in most cases. Dashed vertical lines mark the start and end of P3 bounding windows: 150–300ms for P3a positive peak and 300–1000ms for P3b.

**Figure 10:**
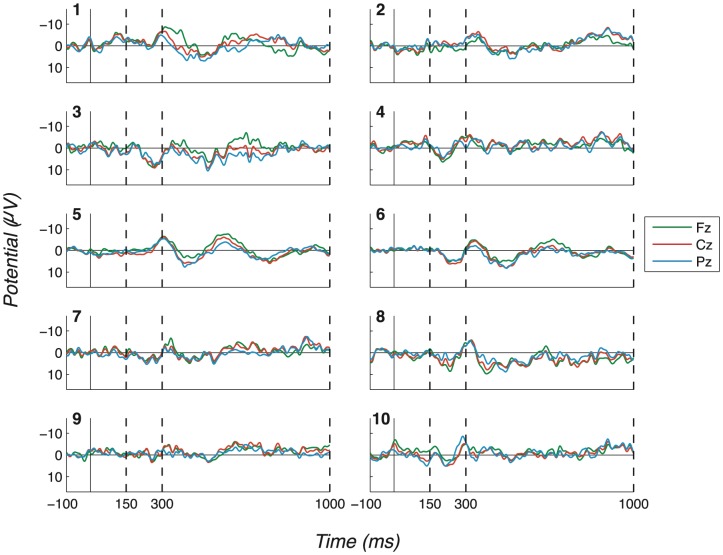
Difference waves for the “Irrelevant as high salient 2” experiment (exp. 4), all channels and participants. (Positive plotted down.) Similarly to Figures 7–9, Probe - Irrelevant1 difference waves for each participant are shown in this figure. Again, we obtained strong P3 responses in most cases, suggesting that this countermeasure strategy did not confound our deception detector. Bounding windows (discussed in Section 2.1.9) are marked by dashed vertical lines (150–300ms for P3a positive peak and 300–1000ms for P3b).

The consistency and robustness of our effects across participants can be seen in the Probe - Irrelevant1 difference waves, on which participant (observed) peak-to-peak values are computed. These are shown in Figure 7 (No countermeasures), Figure 8 (Probe as low salient), Figure 9 (Irrelevant as high salient 1) and Figure 10 (Irrelevant as high salient 2) with P3 bounding regions marked by dashed vertical lines. For most participants, a full-oscillation cycle can be seen at Fz, while a large peak-to-peak oscillation can be seen at Pz. The relative size of positive deflection to following negative deflection varies by participant, but a peak-to-peak difference is clear for all apart from a couple of participants.

#### 3.2.1 Fisher combined analysis

Considering all experiments, Table 4 shows the p-values obtained for each participant in combined 3-dimensional inference, using Fisher scoring. For many participants (15 out of 42), the p-value was smaller than 0.0001; that is, when the three dimensions (P3-Fz, P3-Cz and P3b) were weighed together, there were no null hypothesis data points above the true observed value, clearly indicating presence of those participants’ real name. Twenty-eight have p-values less than or equal to 0.001, and eleven have relatively greater p-values, but still below a 0.05 alpha level, again successfully detecting “own-name” occurrence. The p-value for three participants was above the 0.05 alpha level, two of which were below 0.1 (which is often used as the significance threshold in EEG deception detection [8], [9], [14]), while only one was above the 0.1 threshold.

#### 3.2.2 False positive rate

As previously discussed, we are also interested in the false positive (i.e. Type I error) rate of our overall deception detection approach, over and above the intrinsic false positive rate of our statistical inference method (which was confirmed, as required, to be the alpha level in [22]). This was explored in our Innocents condition (experiment 5). Each of the eight participants saw three Irrelevants, any one of these Irrelevants could play the role of the Probe in our analysis, or, indeed, either of the two Irrelevant roles. Accordingly, to generate a larger number of data points, we analysed 48 data sets, which comprise all the possible allocations of the three Irrelevants to roles (for each participant, there are six allocations of three Irrelevants to roles, since there are six permutations of three items; furthermore, there are eight participants and 6×8 = 48). Out of the 48 null data sets analysed, two yielded significant p-values, see Table 5, which gives no evidence for inflation of the false positive rate, over and above the alpha level. The p-values reported in Table 5 were also used to calculate specificity for the AUC estimates previously reported in Section 3.2.

**Table 5:** Summary table for the empirical false alarm testing procedure applied to the Innocents.

	Permutation no. (p-values)					
Part. No.	1	2	3	4	5	6
1	0.1890	0.6126	0.9795	0.3762	0.8581	0.2679
2	0.5540	0.1789	0.3742	0.3098	0.5384	0.9463
3	0.5916	0.2056	0.3043	0.1039	0.4743	0.4171
4	**0.0337** *	**0.0753**	0.4957	0.7720	0.6576	0.5883
5	0.7921	0.6931	0.6112	0.7730	0.5080	0.4925
6	0.3838	0.1475	**0.0600**	0.1064	0.2395	0.8784
7	**0.0832**	0.3243	0.8332	0.8717	**0.0884**	**0.0094** *
8	0.6265	0.6605	0.8653	0.9486	0.3942	0.5487

* The outcomes that were significant at an alpha level of p<0.05 are indicated with an asterisk (and in bold if p<0.1). Two false alarms are present using an alpha level of 0.05 and six using an alpha level of 0.1, respectively corresponding to false alarm rates of 4.17% and 12.5%.

#### 3.2.3 Questionnaires

We used Fisher’s exact test to compare the amount of Probe recalls between the No countermeasures and Probe as low salient experiments (as described in Section 2.2) and we did not find any evidence that Probe was recalled less frequently in the countermeasure experiment (*p* = 1, left tailed). With respect to the Irrelevant as low salient experiments, the right tailed tests between the No countermeasures and Irrelevant as high salient 1 experiments failed to find any significant increase in the amount of recalls for either Irrelevant1 (*p* = .1053) or Irrelevant2 (*p* = .4286). Similarly, no significant difference was found in the comparison between the No countermeasures and Irrelevant as high salient 2 experiments (*p* = .4286, for both Irrelevant1 and Irrelevant2). A limited number of participants did recall one or two Irrelevants in the Irrelevant as high salient 1 experiment, although this effect was not systematic enough to produce a statistically significant difference. However, a comparison between the Irrelevant as high salient 1 experiment against an hypothetical experiment in which no Irrelevant recalls were reported would produce a significant result. Additional raw behavioural data collected via the recall questionnaires is provided in Tables S4-S9 in Appendix S1).

Wilcoxon rank-sum test results obtained by comparing the scores given by participants to the Noncritical name against the Critical names are presented in Table 6. The only significant differences found are between the Noncritical and Probe/Fake, providing no evidence that participants, in general, assigned higher scores to the Irrelevants. Some participants did, though, assign higher raw confidence scores to the Irrelevants than the Noncritical, although, as just indicated, this effect was not statistically reliable at the group level. Participants’ individual responses are listed in the supplementary material (Tables S10-S11 in Appendix S1).

**Table 6:** Wilcoxon rank sum test results between the responses for the “Noncritical” name in the recognition questionnaires and each “Critical” name.

Experiment	Probe	Fake	Irrelevant1	Irrelevant2
**No Countermeasures**	**0.0002** *	**0.0006** *	0.3869	0.3802
**Probe Low**	**0.0006** *	**0.0015** *	0.3469	0.6860
**Irr High 1**	**0.0001** *	**0.0002** *	0.0088	0.0953
**Irr High 2**	**0.0002** *	0.0025*	0.3159	0.3447
**Innocents**	0.2642	0.0170	0.2129	0.0818

* The outcomes that were significant at an alpha level of p ≤ 0.0025 are indicated with an asterisk (and in bold if p<0.0025).

*Note.* This was a left tailed test, i.e. the alternative hypothesis was that the noncritical name scored lower than the given “critical” name. The significance threshold was set to 0.0025 after applying Bonferroni correction for 20 comparisons. Note that in the Innocents, the Fake was not found significant; this is most likely due to the low number of participants present in that experiment (8).

## Discussion

### 4.1 Irrelevant as high salient experiments

There are a number of points to consider with regard to the Irrelevants as high salient countermeasure. In particular, we did see a change in the Irrelevant ERP and recall/recognition of Irrelevants in experiments three and four, when participants were attempting the Irrelevants as high salient countermeasure. However, this change in the electrophysiological and behavioural pattern was not sufficient to confound the Fringe/P3-Rapid method. Furthermore, the Irrelevants’ P3 pattern remained very different to, even, the Fake pattern. This is important, since a likely consequence of artificially elevating the salience of an Irrelevant is to make it a task-prescribed target, in much the same way as the Fake.

Thus, it would seem that, when participants “see” the Irrelevant, it is sufficiently late in the experiment that any P3 present in those (late) trials is “watered down” when averaged against the earlier P3-absent trials. Note, such late detection of Irrelevants would lead to an increase in recall and recognition of these Irrelevants (which we see in a limited number of cases); after all, our memory tests do take place after all trials have completed. But, such an increase in memory could only reflect very late identification of the Irrelevant.

This, then, certainly supports the statement that “it is *hard* to direct the subliminal search system to detect frequently presented unknown stimuli”. However, it does not necessarily contradict the stronger statement that, “it is *not possible* to direct the subliminal search system to detect frequently presented unknown stimuli”. There are two reasons for this. Firstly, in our experiments, the Irrelevants were selected from a list presented to participants prior to the experimental blocks. This was done to ensure that neither Irrelevant was a priori familiar to the participant; all names marked by the participant as familiar on the pre-experiment list were excluded from the experiment.

Presentation of this pre-experiment list is likely to prime the items included, two of which were the Irrelevants. This may well make it easier for participants to detect the Irrelevants than it might be if they had not been primed and were, thus, completely novel (and, indeed, in a deployed identity detector, one could not perform this pre-experiment screening of items and would, rather, rely on the low probability that an Irrelevant chosen from a list of 3665 names would be highly familiar).

In this sense, our experiment is a rather generous test of the ability to detect stimuli solely on the basis of their frequency (in the sense that detecting stimuli in the memory tests here does not ensure their detectability in a deployed system). Narrower (stricter) tests of this question await further empirical study. It is also, in this respect, in fact a conservative test of our ERP detection method, with regard to the Irrelevants as High Salient countermeasure. Thus, the success of our method at differentiating Probe from Irrelevant ERPs during this countermeasure is very promising.

Secondly, it would certainly seem likely that some RSVP items could be perceived, under what, according to current scientific understanding, would have to be attributed to chance. For example, it could be that, effectively by accident, a name will sometimes occur surrounded by temporally adjacent names that are poor masks for its visual form. As a result, this name may “pop-out” of the stream and be perceived.

The frequency of occurrence of Irrelevants makes them singularly likely to be perceived in this way. Thus, it may be that Irrelevants become “known” to participants through “chance pop-out”. However, the critical difference in the experiment three and four countermeasures cases is that participants are “looking out” for unknown repeating items and, as a result, they recall them more strongly.

Thus, the nature of the perception of Irrelevants really remains an empirical question and it is still possible that direction towards detection of unknown frequent stimuli is, indeed, strictly impossible in subliminal search. However, empirically differentiating between an interpretation based on (at least initially) chance pop-out of Irrelevants or active subliminal search on the basis of their frequency is certainly challenging and, may, in the end, amount to philosophical hair splitting. But, whichever way, in an absolute sense, Irrelevants are being identified, this does not seem to occur quickly enough to provide a workable countermeasure in our RSVP experiments. This is the central point — even when Irrelevants as High Salient countermeasures are performed, we are sill able to robustly distinguish the ERP signature for Probes from that for Irrelevants.

### 4.2 Novelty of Our Approach

This is the first study demonstrating that presenting stimuli on the fringe of awareness impairs perception of non-salient items, hindering countermeasure use. Our approach differs from a related proposal by Lui and Rosenfeld, whose data supported the hypothesis that lie-related stimuli, which are presented subliminally, may differentially affect ERP patterns of subsequent, supraliminal stimuli [18]. In contrast, our hypothesis requires presentation of stimuli at near-subliminal speeds (on the fringe of awareness), allowing perception of salient stimuli, including items which carry concealed knowledge. Specifically, the ERP comparison we are making is between a conscious percept (when the Probe “breaks through” into awareness), and the absence of such a conscious experience (as arises for Irrelevants). The large ERP differences we observe between Probe and Irrelevant seem to reflect this—presence vs. absence of a conscious experience. Electrophysiological responses formed in subliminal priming experiments do not reflect this distinction—the objective in subliminal priming experiments is to render *all* primes subliminal—whether salient (as a Probe is) or non-salient (as an Irrelevant is). Thus, subliminal priming experiments do not induce distinct conscious versus non-conscious states of experience between different classes of prime. Really, the effectiveness of the Fringe/P3-Rapid deception detector rests upon the perceptual regime in which RSVP places the brain; that is, stimuli are presented such that only a small subset of them can be consciously perceived, and, critically, the brain *selects* those to perceive on the basis of their salience. In other words, only salient stimuli break into consciousness and set-up the pronounced electrical response we see in the P3a and P3b.

This study demonstrated high accuracies in the classification of deceivers and non-deceivers based on ERP data alone, even when countermeasures were applied. Previous studies have indeed demonstrated that one’s own name, one of the most over-rehearsed stimuli, can elicit large electrophysiological responses, particularly in frontal regions [33]. The P3a (or novelty P3) was also elicited in comatose patients, by using their own name (as an auditory stimulus) [34]. More generally, the P3a is often elicited when a participant’s own name is presented as a task-irrelevant stimulus [35], [36]. Because of these precedents, we have used the term P3a to identify the early fronto-central oscillations we obtained, although we acknowledge that our P3a patterns differ somewhat from those typical of oddball-type experiments (which are characterised by a slightly later latency and rapid habituation [37]).

The use of first names as stimuli may explain the very high hit rates we obtained, thanks to the strength of our P3a component. Nevertheless, the P3b pattern should be reliable across stimulus types and even if our approach were to show its largest effects in identity deception, it would still be of interest: countermeasure-resistant identity deception is a valuable tool for forensic science (including detection of simulated amnesia). Moreover, P3-based deception detection systems based on the classical oddball paradigm have recently been demonstrated to be vulnerable to countermeasures derived from directed forgetting techniques [38]. The efficacy of such techniques applied to our paradigm remains to be verified.

While confirmation of the full generality of our findings awaits further empirical work, our proposal that the Fringe/P3-Rapid method counters key deception detection countermeasures is supported by the experiments presented here. We argue that our apparent success in subverting countermeasures arises from two key properties of subliminal search.

1. The Probes as low salient countermeasure is countered, since, one cannot direct the search system not to “look” for a stimulus whose identity is intrinsically salient to the individual.
2. The Irrelevants as high salient countermeasure is countered, since volition is impaired in its ability to direct the subliminal search system to “look” for a stimulus without having already (perhaps through task instruction) ascribed salience to its identity.

This leaves us with the summarising adage that during subliminal search, one cannot withdraw salience from the known or impose it upon the not known.

## Supporting Information

Appendix S1 **Additional Data Tables.**
(PDF)

Appendix S2 **Written Instructions.**
(PDF)
