# Supplementary material for: Countering Countermeasures: Detecting Identity Lies by Detecting Conscious Breakthrough
Source: PLoS One. 2014 Mar 7;9(3):e90595. doi: 10.1371/journal.pone.0090595 (PMC3946631; doi:10.1371/journal.pone.0090595)
Supplement: Appendix S1 — Additional Data Tables. (PDF) [file pone.0090595.s001.pdf]

Appendix S1:

Additional Data Tables

This document contains supplementary data collected in the "Countering countermeasures: detecting identity lies by detecting conscious breakthrough" experiments.

End of Stream Answers

Table S1.

Number of times that "Yes" was answered to the question "Did you see your name?" for each trial type.

		Trial Types			
**Group**	**Part. No.**	**Fake**	**Probe**	**Irrelevant1**	**Irrelevant2**
**No C/M (exp. 1)**	1	49	0	4	4
	2	49	1	2	0
	3	39	1	33	38
	4	39	1	3	4
	5	39	1	14	13
	6	49	1	13	9
	7	49	3	50	46
	8	46	1	2	3
	9	21	1	2	1
	10	31	2	17	13
	11	43	2	3	3
	12	38	4	5	4
**Probe Low (exp. 2)**	1	25	2	4	4
	2	47	1	0	3
	3	43	6	6	7
	4	49	0	1	2
	5	34	2	1	2
	6	45	4	11	12
	7	48	0	2	7
	8	41	4	10	10
	9	43	1	1	1
	10	49	1	1	1
**Irr High 1 (exp. 3)**	1	37	2	3	2
	2	42	9	6	8

	3	37	2	2	3
	4	49	3	3	0
	5	35	12	11	18
	6	34	2	1	2
	7	36	1	4	1
	8	39	0	1	0
	9	27	0	1	0
	10	37	0	0	0
**Irr High 2**	1	38	1	0	1
**(exp. 4)**	2	34	8	36	39
	3	45	0	1	1
	4	12	0	4	0
	5	46	0	5	0
	6	44	2	2	1
	7	44	7	2	2
	8	40	3	3	2
	9	42	3	13	5
	10	48	1	7	2
**Innocents***	1	45	2	1	2
**(exp. 5)**	2	48	4	1	3
	3	44	0	1	1
	4	43	2	0	0
	5	47	1	2	5
	6	19	0	0	0
	7	45	3	1	2
	8	46	0	2	3

* In the innocents group, the Probe was not the participant's name but an additional Irrelevant (as described in Section 2.1.3 of the main text).

Note. Participants 3 and 7 in the No countermeasures experiment almost certainly misinterpreted the behavioural instructions and, as a default, responded yes unless they saw their own name. This, though, had no effect on our EEG results. Moreover, following instructions in this particular experiment was not critical, as participants were not required to apply any countermeasure strategy.

Peak-to-Peak Measurements

Table S2.

Peak-to-peak P3 sizes for all participants and conditions (hit rate testing)

		Probe			Irrelevant 2		
**Group**	**Part. No.**	**P3a-Fz**	**P3a-Cz**	**P3b-Pz**	**P3a-Fz**	**P3a-Cz**	**P3b-Pz**
**No C/M**	1	4.1480	4.2387	6.8227	2.7187	2.2804	1.9807
**(exp. 1)**	2	15.5445	13.1127	12.0718	2.3195	1.8564	2.4258
	3	10.6353	11.0307	17.1357	3.6157	0.9653	3.1011
	4	7.9238	8.3625	12.9194	3.6725	5.3629	3.6564
	5	6.2683	9.4447	16.5192	4.1251	4.6692	4.1415
	6	4.1013	3.5963	7.9981	0.5464	-0.4816	1.9246
	7	6.3138	7.3859	18.5804	0.8609	1.2910	2.0734
	8	8.1302	5.6187	13.7261	2.1295	1.8186	3.8736
	9	4.6290	2.7377	7.6746	0.9405	0.8629	4.3808
	10	4.9000	6.3212	20.2312	1.8528	2.5282	5.0415
	11	8.7700	4.6307	19.7231	1.0807	0.6878	4.7571
	12	4.7652	6.5135	0.6387	1.0186	2.8260	2.5370
**Probe Low**	1	7.8820	8.9539	11.5106	1.8338	2.5319	1.3157
**(exp. 2)**	2	7.4055	6.3207	12.1039	3.6305	4.1804	0.8072
	3	4.7768	4.9860	3.4723	4.1120	3.3866	2.6987
	4	6.5072	5.7168	11.0332	2.4899	2.4686	4.1151
	5	4.3947	3.7282	4.0613	2.9175	3.0097	1.5065
	6	7.0050	7.7858	14.5159	2.3740	-0.4255	4.9982
	7	7.6293	7.3961	6.3647	3.1376	1.6694	1.9434
	8	6.7775	5.5401	4.7029	1.9892	1.4850	5.4916
	9	7.9115	8.6000	15.7066	2.8618	3.4896	5.9455
	10	13.1185	11.2283	4.0471	4.1733	0.3162	2.0338
**Irr High 1**	1	4.3224	3.1676	7.4762	3.5562	3.4325	5.3304
**(exp. 3)**	2	3.5516	3.1768	3.0047	2.7139	1.9824	4.2631
	3	8.8814	7.2796	8.4690	4.1576	4.0047	4.1854
	4	8.7457	5.9346	4.6385	4.5493	2.5465	2.2423
	5	6.9473	5.8712	12.3924	3.6338	2.5235	4.9734
	6	9.5862	8.8246	5.1468	3.8007	5.1679	0.2587
	7	9.3280	8.9559	12.1059	4.7171	2.7695	2.7616
	8	6.5459	6.5499	12.5122	3.6887	5.7282	3.6947

	9	11.8899	6.9571	7.7267	5.4123	5.7092	1.5211
	10	7.1042	8.3659	10.6577	-0.4396	-0.1959	4.8429
**Irr High 2**	1	5.6199	2.8507	8.9334	-0.1899	0.1775	1.6830
**(exp. 4)**	2	5.2705	5.0345	9.8764	1.6579	1.9774	3.9068
	3	10.1523	5.5566	7.6260	3.1806	3.1281	6.4097
	4	7.2481	7.6291	4.0566	3.6405	3.3512	4.0610
	5	6.1236	6.0375	7.7117	3.4438	4.7751	2.0258
	6	7.5021	6.6525	6.8739	1.1603	1.3874	2.3679
	7	5.9179	6.8518	6.5440	2.9175	3.1361	1.5706
	8	5.3734	5.4051	5.9108	3.4439	4.2293	3.7181
	9	4.1781	4.6917	4.2306	4.4522	3.3705	0.6460
	10	4.8049	6.6254	5.9726	0.4237	1.3672	4.6040

Note. Probe is consistently larger than Irrelevant 2.

Single-Dimensional and Combined p-values

Table S3.

p-values obtained from the single dimension randomisations and combined three-dimensional Fisher procedure

Group	Part. No.	P3a-Fz	P3a-Cz	P3b-Pz	Fisher*
**No C/M (exp. 1)**	1	0.0225	0.0153	0.0001	0.0008
	2	<0.0001	<0.0001	<0.0001	<0.0001
	3	0.0002	<0.0001	<0.0001	<0.0001
	4	0.0230	0.0042	<0.0001	0.0001
	5	0.0206	<0.0001	<0.0001	<0.0001
	6	0.0232	0.0526	<0.0001	0.0009
	7	0.0105	0.0026	<0.0001	<0.0001
	8	0.0009	0.0436	<0.0001	<0.0001
	9	0.0957	0.3792	0.0002	0.0049
	10	0.0831	0.0166	<0.0001	0.0006
	11	<0.0001	0.0959	<0.0001	0.0001
	12	0.0484	0.0035	0.9350	0.0243
**Probe Low (exp. 2)**	1	0.0021	0.0001	<0.0001	<0.0001
	2	<0.0001	0.0062	<0.0001	<0.0001
	3	0.1718	0.0361	0.2323	0.0761
	4	0.0296	0.0408	<0.0001	0.0004
	5	0.1264	0.1100	0.0665	0.0560
	6	0.0287	0.0008	<0.0001	<0.0001
	7	0.0077	0.0057	0.0263	0.0017
	8	0.0416	0.0475	0.0954	0.0324
	9	0.0005	0.0004	<0.0001	0.0001
	10	<0.0001	<0.0001	0.4431	<0.0001
**Irrelevant High 1 (exp. 3)**	1	0.1345	0.2888	0.0062	0.0302
	2	0.3799	0.4044	0.5566	0.4919
	3	0.0002	0.0014	0.0007	0.0001
	4	<0.0001	0.0034	0.0270	0.0002
	5	0.0084	0.0246	<0.0001	0.0006
	6	<0.0001	<0.0001	0.0565	<0.0001
	7	0.0006	0.0001	<0.0001	<0.0001
	8	0.0054	0.0022	<0.0001	<0.0001

	9	0.0002	0.0465	0.0431	0.0005
	10	0.0004	0.0001	0.0001	<0.0001
**Irrelevant High 2 (exp. 4)**	1	0.1448	0.5338	0.0001	0.0057
	2	0.0238	0.0308	<0.0001	0.0007
	3	<0.0001	0.0405	0.0005	<0.0001
	4	0.0058	0.0044	0.2580	0.0048
	5	0.0039	0.0065	0.0002	0.0002
	6	<0.0001	<0.0001	0.0001	<0.0001
	7	0.0337	0.0055	0.0179	0.0037
	8	0.0618	0.0548	0.0236	0.0181
	9	0.0752	0.0482	0.1095	0.0397
	10	0.1019	0.0041	0.0330	0.0070

* Fisher combined probabilities, which are the main indicator of concealed information in our analysis, are summarised in Table 4 of the main text.

Recall Questionnaire Answers

Table S4.

Recall table for all experiments except the Innocents

Group	P. No.	Fake	Probe	Irr. 1	Irr. 2	Group	P. No.	Fake	Probe	Irr. 1	Irr. 2
**No C/M**	1	X	X			**Irr High 1**	1	X	X		
**(exp. 1)**	2	X	X			**(exp. 3)**	2	X	X		
	3	X	X				3			X	X
	4	X					4	X	X	X	
	5	X	X				5	X	X		
	6	X	X				6	X			
	7	X	X	X			7	X	X		
	8	X	X				8	X	X	X	
	9	X					9	X	X	X	X
	10	X	X				10	X	X		
	11	X	X		X						
	12	X									
**Probe Low**	1	X	X			**Irr High 2**	1	X	X		
**(exp. 2)**	2	X	X			**(exp. 4)**	2		X		
	3	X	X				3	X	X		
	4	X	X				4	X			
	5	X	X				5	X	X	X	
	6	X	X				6	X	X		
	7	X	X				7	X	X		
	8	X	X				8	X	X		X
	9	X	X				9	X	X	X	
	10	X	X				10	X	X		X

Note. An ‘X’ in this table indicates that the given participant wrote the corresponding critical item in the Recall questionnaire. There is a slight increase in the number of times that the Irrelevants were recalled by participants in the Irrelevant as high salient experiments (6 and 4 against 3 on average). However, this did not greatly affect our hit rate (9/10) or p-values (e.g. p-values for participants 3 and 9, experiment 3 were all below 0.05, despite both Irrelevants being successfully recalled—these data were previously presented in Table S3 in Appendix S1).

Table S5.

Recall table for the Innocents

**Part. No.**	**Fake**	**Irrelevant1**	**Irrelevant2**	**Irrelevant3**
1	X			
2	X			
3	X			
4	X	X		
5	X			
6				
7	X	X	X	
8	X	X		

Table S6.

Contingency table for Probe, "No countermeasures" (exp. 1) against "Probe as low salient" (exp. 2)

	**Recalled**	**Not recalled**	**Total**
**No C/M**	9	3	12
**Probe Low**	10	0	10
**Total**	19	3	22

Note. Fisher's exact test returns p=1 (left tailed). This reflects the probability that Probe was not recalled *less* times in the countermeasure condition. In other words, there is no evidence that participants in the countermeasure condition recalled their name less frequently.

Table S7.

Contingency table for Irrelevant1, "No countermeasures" (exp. 1) against "Irrelevant as high salient 1" (exp. 3)

	**Recalled**	**Not Recalled**	**Total**
**No C/M**	1	11	12
**Irr High 1**	4	6	10
**Total**	5	17	22

Note. Fisher's exact test returns p=.1053 (right tailed). In this case, we tested whether participants were able to recall the irrelevants *more often* in the countermeasure condition. The test failed to reject the null hypothesis that there was no such difference.

Table S8.

Contingency table for Irrelevant2, "No countermeasures" (exp. 1) against "Irrelevant as high salient 1" (exp. 3)

	**Recalled**	**Not Recalled**	**Total**
**No C/M**	1	11	12
**Irr High 1**	2	8	10
**Total**	3	19	22

Note. Fisher's exact test returns p=.4286 (right tailed).

Table S9.

Contingency table for both Irrelevants (which were identical), "No countermeasures" (exp. 1) against "Irrelevant as high salient 2" (exp. 4)

	**Recalled**	**Not Recalled**	**Total**
**No C/M**	1	11	12
**Irr High 2**	2	8	10
**Total**	3	19	22

Note. Fisher's exact test returns p=.4286 (right tailed). Since Irrelevant1 stimuli were recalled exactly the same number of times as Irrelevant2 stimuli, this contingency table covers both Irrelevants.

Recognition Questionnaire Answers

Table S10.

Recognition table, for all experiments except the Innocents

Group	Part. No.	Fake	Probe	Irrelevant1	Irrelevant2	Noncritical
**No C/M (exp. 1)**	1	4	4	2	3	3
	2	4	5	1	3	2
	3	4	4	3	2	1
	4	4	4	2	1	2
	5	3	4	2	1	2
	6	4	4	2	2	1
	7	3	3	2	1	2
	8	3	4	2	1	2
	9	1	4	3	2	3
	10	3	4	2	3	1
	11	3	5	1	2	1
	12	4	1	2	5	3
	**AVERAGE**	**3.3**	**3.8**	**2**	**2.2**	**1.9**
**Probe Low (exp. 2)**	1	3	5	3	3	2
	2	3	4	4	1	1
	3	4	3	1	2	1
	4	5	4	3	1	4
	5	3	3	4	2	3
	6	4	4	3	2	2
	7	4	4	1	3	3
	8	5	4	2	2	3
	9	5	5	2	3	2
	10	3	4	2	2	2
	**AVERAGE**	**3.9**	**4**	**2.5**	**2.1**	**2.3**
**Irr High 1 (exp. 3)**	1	4	4	2	2	1
	2	3	4	4	1	2
	3	3	3	2	5	2
	4	5	5	3	1	1
	5	3	3	2	2	1
	6	4	4	3	2	1
	7	4	5	4	5	3

	8	4	5	2	4	2
	9	4	4	5	4	3
	10	5	5	2	1	1
	**AVERAGE**	**3.9**	**4.2**	**2.9**	**2.7**	**1.7**
**Irr High 2 (exp. 4)**	1	5	4	1	2	2
	2	2	3	1	2	2
	3	5	4	1	3	2
	4	3	5	4	1	2
	5	2	4	5	1	4
	6	4	5	2	4	1
	7	4	5	2	3	2
	8	4	4	4	3	2
	9	5	5	3	1	3
	10	4	3	3	5	2
	**AVERAGE**	**3.8**	**4.2**	**2.6**	**2.5**	**2.2**

Note. At the end of each experiment, after the recall questionnaire, participants were asked to rate a given set of 5 names for familiarity. They were asked to do so with a number ranging from 1 to 5, where 5 indicated that they saw the name frequently and 1 indicated that they did not see the name. The Noncritical did not appear often in the experiment (perhaps once or twice). It was included in the questionnaire as a simple means to assess the response bias of participants. Interestingly, recognition performance on Irrelevants was higher than on Noncritical names (2.4 instead of 2.0, on average), although considerably less than on Fakes or Probes (which obtained a score of 3.9 on average). Thus, while a distinct recallable representation of Irrelevants does not seem to reliably manifest in memory, some form of pre-memory encoding representation looks to be present. Though requiring more experimental study, this pattern for Irrelevants suggests a form of subliminal priming, which has been argued to obtain in the RSVP context (Rolke et al., 2001; Pesciarelli et al., 2007). It also chimes with the distinction between tokenised stored representations (which would be recallable) and earlier, item layer, representations (which might facilitate recognition) in the simultaneous type / serial token model (Bowman and Wyble, 2007; Wyble et al., 2009); and even with the distinction between access (recallable) and phenomenological (perhaps facilitating

recognition) awareness (Block, 2007).

Table S11.

Recognition table for the Innocents

**Part. No.**	**Fake**	**Irrelevant1**	**Irrelevant2**	**Irrelevant3**	**Noncritical**
1	2	1	3	2	3
2	4	3	5	3	1
3	3	2	2	4	1
4	5	3	4	3	1
5	3	1	4	2	2
6	3	4	1	2	5
7	3	3	1	2	2
8	4	2	2	4	2
**AVERAGE**	**3.375**	**2.375**	**2.75**	**2.75**	**2.125**

References

Bowman, H., & Wyble, B. (2007). The simultaneous type, serial token model of temporal attention and working memory. *Psychological Review*, *114*(1), 38-70.

Rolke, B., Heil, M., Streb, J., & Hennighausen, E. (2001). Missed prime words within the attentional blink evoke an N400 semantic priming effect. *Psychophysiology*, *38*(2), 165-174.

Pesciarelli, F., Kutas, M., Dell'Acqua, R., Peressotti, F., Job, R., & Urbach, T. P. (2007). Semantic and repetition priming within the attentional blink: An event-related brain potential (ERP) investigation study. *Biological psychology*, *76*(1-2), 21-30.

Wyble, B., Bowman, H., & Nieuwenstein, M. (2009). The attentional blink provides episodic distinctiveness: Sparing at a cost. *Journal of Experimental Psychology: Human Perception and Performance*, *35*(3), 787-807.

Block, N. (2007). Consciousness, accessibility, and the mesh between psychology and neuroscience. *Behavioral and Brain Sciences*, *30*(5-6), 481-548.
