# Supplementary material for: Countering Countermeasures: Detecting Identity Lies by Detecting Conscious Breakthrough
Source: PLoS One. 2014 Mar 7;9(3):e90595. doi: 10.1371/journal.pone.0090595 (PMC3946631; doi:10.1371/journal.pone.0090595)
Supplement: Appendix S2 — Written Instructions. (PDF) [file pone.0090595.s002.pdf]

Appendix S2:

Written Instructions

Basic Instructions

The basic written instructions that were given to all participants in all groups were as follows:

- Sit comfortably in the chair and relax; EEG signals are adversely affected by muscle movements and muscle tension.
- During this experiment, you will be presented a series of trials. Each trial will consist of a fast succession of stimuli (names).
- The last item of every trial will not be a name. It will be either '=======' or '-------'. It will be followed by the question 'Which one of the two possible last items did you see?' Answer the truth to that question.
- Remember that your task is to LIE about your name. Answer YES to the "did you see your name?" question only if you saw your FAKE name. Answer NO if you saw your real name.
- Also answer NO to the question if you saw neither the FAKE nor real name.
- Try not to blink during a trial. You can blink and relax during the questions, or before pressing ENTER to proceed with the experiment.
- You should answer the questions quickly, by making your best guess.
- Wait for the trial to finish before moving your hands.
- If your eyes feel dry, take a short break before continuing with the experiment. It may help to blink during this break.
- If there is anything wrong, you can stop the experiment at any time by raising your hand: there is a camera in the lab and I will see you.

Additional Instructions

In addition to the basic instructions just listed, we gave participants an additional set of instructions depending on the countermeasure group that they were assigned to (if any).

Additional Instructions: Probe as Low Salient

- The lie detector works by detecting that you "see" your real name. Your task is to fool the lie detector.
- You should concentrate hard on "seeing" the FAKE and not "seeing" your real name.

Additional Instructions: Irrelevant as High Salient 1

- In addition to the FAKE and your real name, two further names appear frequently in the experiment. You should attempt to "see" these names, in order to count how often each occurs during the experiment.
- You should, though, still respond NO to the "did you see your name?" question on all trials apart from those in which you see your FAKE name. So, only respond YES to the FAKE.

Additional Instructions: Irrelevant as High Salient 2

- In addition to the FAKE and your real name, two further names appear frequently in the experiment. In order to fool the lie detector, you should attempt to "see" these names. If you manage to see one of them, pretend it is your REAL name.
- You should, though, still respond NO to the "did you see your name?" question on all trials apart from those in which you see your original FAKE name. So, only respond YES to the original FAKE name.
